# The Qualification of Outcome after Cervical Spine Surgery by Patients Compared to the Neck Disability Index

**DOI:** 10.1371/journal.pone.0161593

**Published:** 2016-08-23

**Authors:** Roland Donk, Andre Verbeek, Wim Verhagen, Hans Groenewoud, Allard Hosman, Ronald Bartels

**Affiliations:** 1 Department of Orthopedic Surgery, Via Sana Clinics, Hoogveldseweg 1, 5451 AA, Mill, the Netherlands; 2 Department for Health Evidence, Radboud university medical center, Geert Groote Plein-zuid 10, 6525 GA, Nijmegen, the Netherlands; 3 Department of Neurology, Canisius Wilhelmina Hospital, Weg door Jonkerbos 100, 6532 SZ, Nijmegen, the Netherlands; 4 Department for Orthopedic Surgery, Radboud university medical center, Geert Groote Plein-zuid 10, 6525 GA, Nijmegen, the Netherlands; 5 Department of Neurosurgery, Radboud university medical center, Geert Groote Plein-zuid 10, 6525 GA, Nijmegen, the Netherlands; 6 Canisius Wilhelmina Hospital, Department of Neurosurgery, Weg door Jonkerbos 100, 6532 SZ, Nijmegen, the Netherlands; Universita degli Studi di Palermo, ITALY

## Abstract

**Objective:**

The Neck Disability Index (NDI) is a patient self-assessed outcome measurement tool to assess disability, and that is frequently used to evaluate the effects of the treatment of neck-related problems. In individualized medicine it is mandatory that patients can interpret data in order to choose a treatment. A change of NDI or an absolute NDI is generally meaningless to a patient. Therefore, a correlation between the qualification of the clinical situation rated by the patient and the NDI score was evaluated.

**Methods:**

Patients who completed an NDI after anterior surgery because of symptomatic single level degenerative cervical disc disease were asked one month after completion of the NDI to qualify their clinical situation of a 5-item Likert scale varying from excellent to bad. Since a clear distinction between the categories was not possible based on the total NDI score, a ROC-curve was built, and the AUC computed in order to estimate best dichotomization in qualification of the clinical situation. The best corresponding cut-off point for the NDI total score was found by studying sensitivity and specificity for all possible cut-off points.

**Results:**

102 patients were included. The highest AUC was obtained by dichotomizing the qualification into a group with good outcome and less-good outcome. The highest sensitivity and specificity for the dichotomized qualification as good outcome corresponded to a NDI ≤ 7. Sensitivity was 81.08% and specificity was 78.57%.

**Conclusion:**

This is the first study that correlated the qualification of the situation by the patients themselves and NDI. An NDI ≤ 7 corresponded to a good outcome according to the patients. This is valuable information to inform patients in their decision for any treatment.

## Introduction

The Neck Disability Index (NDI) is a frequently used, well known, and in multiple languages validated outcome measurement tool to assess self-rated disability in patients with neck pain. It can be categorized as a patient reported outcome measurement tool (PROM). The NDI is frequently used in clinical practice, but also for research purposes [1–3]. The main purpose is the quantification of the difference in pre- and post-treatment condition according the patients suffering from disabling neck pathology. The NDI addresses pain and functional items related to neck problems. It has been validated in both neck pain and, especially, whiplash patients [2, 4].

Informing the patient is crucial before installing any treatment. In modern times information can be gained through many resources, but the treating physician is still very important. It has been shown that fulfillment of preoperative expectations is related to the highest post-operative satisfaction. A mismatch of disease understanding and expectation between treating physician and patient might result is a less than favorable outcome according to the patient [5–7].

The information provided by PROMs as the NDI obtained from studies can contribute in sketching expectations while informing the patient before any treatment. The most useful tools in the process of gaining information or providing it are clear clinical outcomes: mortality, infection rate etc. However, PROMs including the NDI are not reporting on a clearly defined outcome but on a combination of surrogate outcomes.

An adequate interpretation of a PROM is difficult, especially since it has been demonstrated that the language used in the questionnaires is very difficult to understand for patients. As El-Daly,I. et al. stated in their conclusion: “the majority of PROMs analyzed are written at a level that is incomprehensible to the average UK adult”[8]. The usefulness of the results of PROMs with low readability is debatable.

However, the NDI was also incorporated in the earlier mentioned study. For a correct understanding of the NDI an education level of 13–15 year-old subject was needed indicating a readability level of standard English [8]. Since the translation of the NDI into Dutch has been validated [9] we feel confident that most of the patients did understand the questions and completed their questionnaires without difficulty.

Although it seemed related, the readability of a PROM is different than interpreting the result. For example and specifically for the NDI, what information is provided to the patient if he reads or hears that a mean total score of the NDI of 8 is achieved in a group of 100 patients after a certain treatment. Information should be presented in a way that is acceptable and useful for a patient [10]. In a survey among patients with scoliosis and their carers, it was advised that the information should be user friendly and in plain language [11]. For NDI grades of disability have been defined, although these also differ and are based on clinical information and not the patients qualification [2]. A grade of disability like “none to mild disability” is not very illustrative to a patient.

Therefore, we would like to correlate the total score of the NDI with a qualitative rating by the patient themselves in a way that everyone can understand. This will contribute to understanding and decision making for patients in the future.

## Methods

The STROBE statement was followed (S1 File)[12]. The ethical board CMO Arnhem-Nijmegen approved the study. The study has been carried out in accordance with the World Medical Association Declaration of Helsinki [13].

Patients who participated in the Procon trial Current (Controlled Trials ISRCTN41681847) [14], a comparison of different anterior cervical surgery techniques for symptomatic single level degenerative disc herniation without spinal cord involvement, and who completed a NDI were included. 142 patients participated of whom 140 completed and returned the NDI. One patient died unrelated to the trial, the other refused to return the NDI questionnaire. So, 140 patients were eligible. The mean time after surgery was 9.1 ± 1.9 years (5.6–12.2 years).

Within two months after completion of the NDI, a questionnaire was sent to the patients about the qualification of their situation regarding the neck and its related problems at that moment. Although little is known about the bias introduced by sending reminders [15], we did not send reminders or contacted the non-responders.

A five-item Likert scale was used. We did not predefine the criteria, since we were interested in the qualitative judgment of the patients themselves without any bias introduced by the researcher. The possible qualifications of their situation were: excellent, very good, good, moderate, and bad (see S2 and S3 Files).

For statistical analyses SAS version 9.2 (SAS Institute Inc. Cary NC, USA) was used. Continuous variables are depicted as value ± standard deviation (minimum-maximum). For data analysis the Student-t test was used. Dichotomization of the patient qualifications seemed to be appropriate. To estimate which qualifications could be best combined for each possible dichotomized set, a ROC curve was build and the area under the curve (AUC) was calculated. The combination with the highest AUC was chosen. To estimate the value of NDI that corresponded best with the dichotomized outcome, the cut-off value of the total NDI with the highest sensitivity and specificity was chosen. A P value < 0.05 was assumed to be statistically significant.

## Results

**Fig 1:**
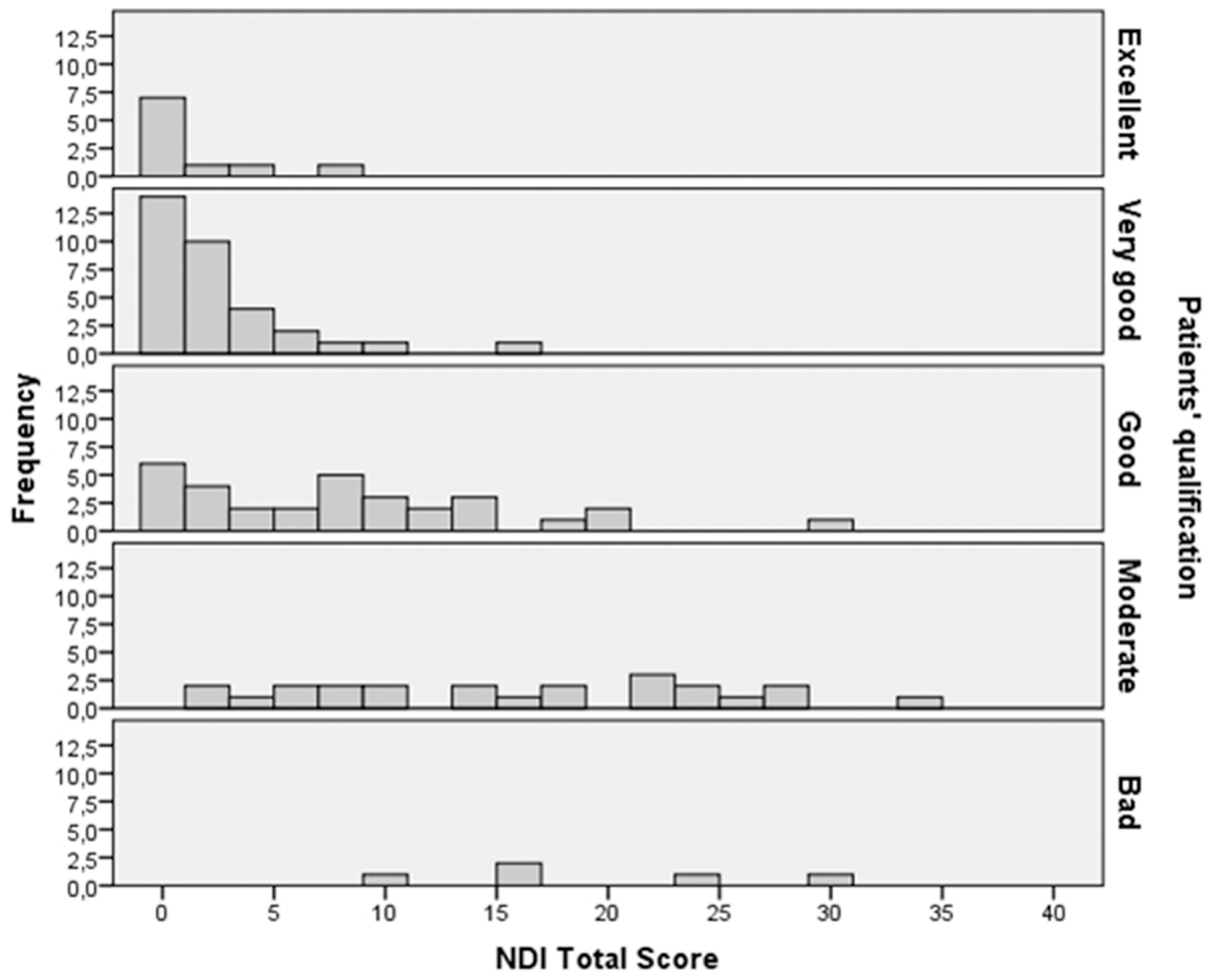
distribution of total ND score in relation to patients’ qualification.

Of the 140 eligible patients, 102 consecutive patients completed the questionnaires (response rate: 72.9%). Mean NDI was 7.5 ± 8.6 (0–34) for the responders and 6.7 ± 8.3 for non-responders. The difference in NDI did not reach statistical significance (P = 0.6). Ten patients rated their situation excellent, 33 very good, 32 good, 23 moderate, and 5 qualified their situation as bad. 73.5% of the patients rated their situation as good or better. In Fig 1 NDI is represented in relation to the Likert qualification. It was not possible to distinct the qualifications clearly based on a total NDI score. Therefore, we decided to dichotomize qualification by the patient.

The biggest AUC was obtained by dichotomizing the qualifications in the group excellent, very good and good versus the combination of moderate and bad (AUC = 0.874). The first group consisted of those patients with a good outcome; the patients belonging to the latter will be regarded as having a less-good outcome.

**Fig 2:**
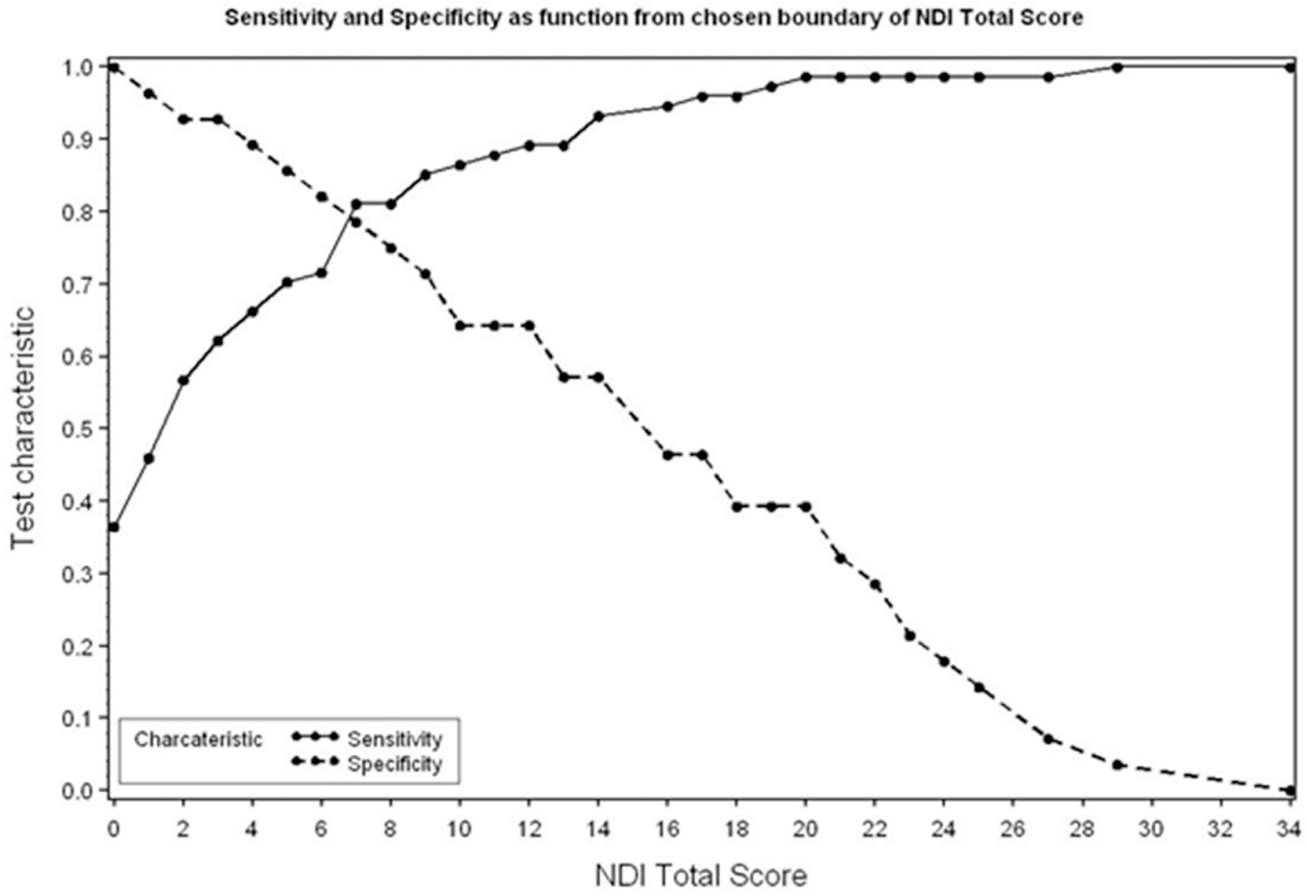
Figure depicting the cut-off value of the total NDI with the highest sensitivity and specificity.

Then a ROC was constructed (Fig 2). The highest sensitivity and highest specificity for a good outcome is obtained when NDI is seven or less: sensitivity was 81.08% and specificity was 78.57%. The distribution of patients after dichotomization in relation to the NDI is shown in Table 1.

**Table 1:** Distribution of patients based on outcome defined as good or less-good in relation to NDI.

	Good	Less-Good	Total
NDI ≤ 7	60	14	74
NDI > 7	6	22	28
Total	66	46	102

## Discussion

Currently, information about any treatment is very easy accessible to patients. However, interpretation of the data is very difficult or even impossible for most patients due to lack of adequate knowledge. Surrogate outcomes are provided that are valuable for scientific purposes, but are not easily transposed to lay terms.

The NDI is a questionnaire assessed by the patient self. The NID consists of ten questions, and for each question six answers are possible. The answers are ordered starting from no disability to maximal disability. The answers are graded from zero to six, and therefore the total NDI score can vary between zero and fifty. The best outcome will be a total score of zero.

The NDI has not been uniformly divided in grades of disability [16–18]. A major concern is furthermore that the investigators predefined the qualifications of each grade. They correlated it to existing questionnaires or findings at physical examinations.

From an investigators point of view a total score of the NDI of zero would correspond with an excellent outcome. We have shown that only a proportion of the patients that rated their situation as excellent had a total NDI score of zero, whereas some patients that rated their situation as good or very good, also had a total NDI score of zero. Other (probably psychological) factors that are not taken into account in the NDI, might explain this.

Transforming a total NDI score into an expression that can easily be understood by patients will help them in making a decision about their eventual treatment, and is a contribution to individualized medicine. This is achieved not only by calculating a cut off value for the total NDI score (NDI ≤ 7 versus NDI > 7), but also by dichotomizing the patients’ qualification in good and less good.

Not actively motivating patients to respond might be considered a flaw of the study. However, comparison of the NDI between the group of responders and non-responders convinced us that the sample is representative. Especially when the response rate of more than 70%, that can be considered as good [19], is taking into account.

Another limitation of the study could be the lack of a pre-inquiry definition of the qualifications as rated by the patients. Therefore, the distribution of the NDI for any qualification is much wider than when the qualifications were defined prior to asking the patients. However, this would have been again the interpretation of the researcher, whereas at this moment we are convinced that the qualifications really represented the perspective of the patient.

Finally, determining the cut off value of the NDI to consider a good or less good outcome can be subject of debate. We have chosen for a conservative approach by requesting the highest sensitivity in combination with the highest specificity. Increasing the NDI score would increase sensitivity and decrease specificity, and decreasing the NDI would induce a reverse effect creating, in our opinion, a less reliable definition of good and less outcome.

Although we did not investigated whether the patients have a better understanding of the expression of a good outcome compared to mild disability, we are convinced that the first is more appealing. From a patients perspective a total NDI score or a difference in NDI score, that is however important for scientific evaluation, is meaningless. It will not help him/her in decision-making about any treatment for neck-related problems.

In conclusion, to help the patient in the decision-making for any treatment of neck-related pathology it seems obvious that expressions should be used that are understandable. Therefore, we propose that a NDI of seven or less is qualified as a good outcome.

## Supporting Information

S1 File STROBE 2007 (v4) Statement—Checklist of items that should be included in reports of *cohort studies*. (DOCX)

S2 File NDI original data. (XLSX)

S3 File Questionnaire translated into English. (DOCX)
