# Supplementary material for: The Qualification of Outcome after Cervical Spine Surgery by Patients Compared to the Neck Disability Index
Source: PLoS One. 2016 Aug 23;11(8):e0161593. doi: 10.1371/journal.pone.0161593 (PMC4995029; doi:10.1371/journal.pone.0161593)
Supplement: S3 File — (DOCX) [file pone.0161593.s003.docx]

**Supporting information file**

**S3 file: Questionnaire translated into English**

Question: How do you rate your current situation regarding your neck ?

1. excellent
2. very good
3. good
4. moderate
5. bad
